# Positive association between Interleukin-8 -251A > T polymorphism and susceptibility to gastric carcinogenesis: a meta-analysis

**DOI:** 10.1186/1475-2867-13-100

**Published:** 2013-10-22

**Authors:** Daye Cheng, Yiwen Hao, Wenling Zhou, Yiran Ma

**Affiliations:** 1 Department of Transfusion, First Hospital of China Medical University, North Nanjing Street, No. 155, Shenyang 110001, Liaoning, PR China

**Keywords:** Interleukin-8, Gastric cancer, Precancerous lesions, Meta-analysis

## Abstract

**Backgrounds:**

The associations between the polymorphisms of interleukin-8 (IL-8) gene and gastric carcinogenesis have been extensively investigated in recent years. However, the results remain conflicting rather than conclusive.

**Methods:**

A meta-analysis of 18 eligible studies was performed to evaluate the association of IL-8 -251A > T polymorphism with risk of gastric carcinogenesis. A systematic literature search of MEDLINE, Embase, and Web of Science, CNKI databases was conducted. Statistical analysis was performed by using the Revman 5.1 software and the Stata 12.0 software.

**Results:**

Of the 293 unique studies identified using our search criteria, 18 studies fulfilled our inclusion criteria and were included in the meta-analysis. These studies cumulatively reported 5,321 cases and 6,465 controls. The combined results based on all studies showed that the IL-8 -251A > T polymorphism was associated with the risk of gastric carciongenesis (A vs. T: OR: 1.14 [1.02, 1.26], *P* = 0.02), especially gastric cancer (A vs. T: OR: 1.15 [1.03, 1.29], *P* = 0.02), but not associated with the risk of precancerous lesion (A vs. T: OR: 1.09 [0.99, 1.20], *P* = 0.08). Analysis stratified by ethnicity may seem that IL-8 -251A > T polymorphism was susceptible to gastric cancer in Asian population, but not in Caucasian population.

**Conclusions:**

Our meta-analysis results provide evidence that IL-8 -251A > T polymorphism is significantly associated with increased risk of gastric carcinogenesis in Asian population, particularly in gastric cancer. Further large and well-designed studies are required to confirm this conclusion.

## Backgrounds

Although the incidence of gastric cancer has recently declined in several countries, it is still a serious health problem and remains the world’s fourth common malignancy and the second leading cause of cancer death [1-3]. Currently, many epidemiologic studies have demonstrated that gastric cancer has a multifactorial etiology and is co-modulated by different factors including *Helicobacter pylori* infection, life style, socioeconomic status, and environmental factors [4]. In addition, genetic factors are increasingly recognized as major contributors to gastric cancer risk [5], although not yet well understood. Perhaps, the genes involved in gastric cancer mutated which intimately control cell growth and apoptosis, allowing cells to acquire the ability to invade and metastasize. Therefore, identification of biomarkers significantly related to development and progress of gastric cancer and elucidation of the molecular mechanisms for cancer prevention and control strategy are essential for better gastric treatment.

Chemotactic cytokines, produced by tumor and endothelial cells, could play an important role in cancer, such as increasing angiogenesis, stimulating tumor progression, enhancing tumor cell migration, and facilitating evasion of immune surveillance [6,7]. As a member of the chemokine family, interleukin-8 (IL-8) is well known for its leukocyte chemotactic properties and its tumorigenic and proangiogenic activities [8]. In vivo and in vitro experiments in melanoma [9], as well as breast [10], ovarian [11], prostate [12], endometrial [13], and colon cancer [14] have shown a direct correlation between IL-8 levels and tumor progression [8]. Moreover, it has been reported that expression of IL-8 in gastric cancer specimens was significantly higher than in corresponding normal gastric mucosa [15], and is associated with adhesion, migration and invasion in gastric cancer [15]. Therefore, it is reasonable to deduce that IL-8 plays a certain role in the formation and progression of gastric tumor.

IL-8 gene, which is located on chromosome 4q12-21, contains four exons and three introns, and exhibits functional polymorphisms, fifteen of which have been characterized [16]. Among these polymorphisms the presence of IL-8-251 A > T in the promoter region exerts the greatest influence on IL-8 production and is associated with the risk of prostate [17], breast [18], oral [19], colorectal cancer [20] and Kaposi’s sarcoma [21]. To better understand this issue, we performed an updated systemic review and meta-analysis of all eligible case–control studies to provide insights into the association between IL-8 -251A > T polymorphism and susceptibility to gastric carcinogenesis, which may promote our understanding of the exact role of IL-8 gene in the etiology of gastric cancer.

## Results

### Search results

**Figure 1:**
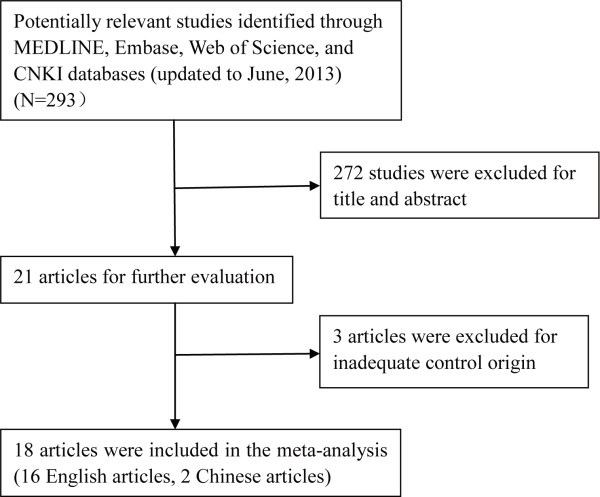
**Flow chart of study selection.** (CNKI, Chinese National Knowledge Infrastructure).

Of the 293 unique studies identified using our search criteria, 18 case–control studies fulfilled our inclusion criteria and were included in the meta-analysis [22-39]. These studies cumulatively reported 5,321 cases and 6,465 controls. The publication year of involved studies ranged from 2004 to 2012. Detailed search steps were described in Figure 1.

### The characteristics of included studies

The main features of the studies included in the meta-analysis were shown in Table 1. Among these studies, six studies were performed in Caucasian populations [22-25,31,32], eleven studies in Asian populations [26-29,33-35,37-39], and one study in mixed population [36]. Ten studies used hospital-based controls, while the other eight studies used population-based controls (community populations). Most studies indicate that the distribution of genotypes in controls was consistent with Hardy–Weinberg equilibrium (HWE), and the subjects of controls were matched for age and gender. All cases were confirmed histologically or pathologically.

**Table 1:** Baseline characteristics of the 18 eligible studies for the analysis of IL-8-251 A > T polymorphism

**Studies**	**Year**	**Country**	**Ethnicity**	**Source of controls**	**Number of controls**	**Number of case**	**Genotyping method**	**HWE**
Burada F	2012	Romanian	Caucasian	HB	242	105	Real-time PCR	0.386
Canedo P	2007	Portugal	Caucasian	PB	693	401	Taqman	0.460
Crusius JB	2008	European	Caucasian	HB	1139	428	Real-time PCR	0.706
Kamangar F	2006	Finland	Caucasian	PB	207	112	Taqman	0.055
Kang JM	2009	Korea	Asian	HB	322	645	RFLP	0.226
Lee WP	2005	Taiwan	Asian	HB	308	461	RFLP	0.143
Liu J	2009	China	Asian	HB	137	417	Taqman	0.145
Lu W	2005	China	Asian	PB	300	250	DHPLC	0.516
Ohyauchi M	2005	Japan	Asian	PB	346	212	Direct sequence analysis	0.549
Savage SA	2004	Poland	Caucasian	PB	429	88	SBE	0.885
Savage SA	2006	Poland	Caucasian	PB	428	287	Taqman or MGB Eclipse	0.391
Shirai K	2005	Japan	Asians	HB	468	181	RFLP	0.830
Song B	2010	China	Asian	HB	190	208	RFLP	0.389
Taguchi A	2005	Japan	Asian	HB	252	611	RFLP	0.994
Vinagre RM	2011	Brazil	Mixed	HB	103	102	RFLP	0.151
Ye BD	2009	Korea	Asian	HB	206	399	RFLP	0.553
Zeng ZR	2005	China	Asian	PB	196	206	PCR-RDB	0.022
Zhang LW	2010	China	Asian	PB	504	519	PCR-RFLP	0.754

*PB*, population-based controls; *HB*, hospital-based controls. *HWE*, Hardy–Weinberg equilibrium. *PCR*, polymerase chain reaction; *RFLP*, restriction fragment length polymorphism; *SBE*, single base extension; *PCR-RDB*, polymerase chain reaction- reverse dot blot.

### Quantitative data synthesis

Table 2 showed the summary odds ratio (OR) relating IL-8-251 A > T to gastric carcinogenesis risk based on 5,321 cases and 6,465 controls in all 18 studies.

**Table 2:** Main results for the IL-8-251 A > T polymorphism with the risk of gastric carcinogenesis based on OR and 95% CI

**Genotype comparison**	**OR [95% CI]**	**Z (*P*value)**	**Heterogeneity of study design**		**Model**
			**χ**^**2**^	**I**^**2**^	
**Overall analysis (5,321 cases, 6,465 controls)**					
A allele vs T allele	1.14 [1.02, 1.26]	2.33 (0.02)	59.55	71%	Random
Asian	1.20 [1.06, 1.36]	2.87 (<0.01)	29.42	66%	Random
Caucasian	0.95 [0.85, 1.07]	0.87 (0.38)	7.38	32%	Random
AA vs AT + TT (dominant model)	1.17 [0.98, 1.38]	1.78 (0.07)	40.15	58%	Random
Asian	1.28 [1.02, 1.61]	2.11 (0.04)	24.53	59%	Random
Caucasian	0.92 [0.79, 1.08]	1.02 (0.31)	6.42	22%	Fixed
AA + AT vs TT (recessive model)	1.18 [1.02, 1.36]	2.21 (0.03)	50.59	66%	Random
Asian	1.26 [1.08, 1.47]	2.86 (<0.01)	23.13	57%	Random
Caucasian	0.90 [0.78, 1.03]	1.53 (0.13)	5.29	70%	Random
AA vs TT (homozygous comparison)	1.26 [1.02, 1.57]	2.10 (0.04)	52.95	6%	Fixed
Asian	1.40 [1.08, 1.83]	2.50 (0.01)	27.43	64%	Random
Caucasian	0.87 [0.73, 1.05]	1.48 (0.14)	7.62	34%	Fixed
AT vs TT (heterozygous comparison)	1.14 [1.00, 1.31]	1.97 (0.05)	39.34	57%	Random
Asian	1.21 [1.09, 1.35]	3.55 (<0.01)	18.92	47%	Random
Caucasian	0.91 [0.78, 1.05]	1.29 (0.20)	3.64	0%	Fixed
**Gastric cancer analysis (4,513 cases, 6,465 controls)**					
A allele vs T allele	1.15 [1.03, 1.29]	2.43 (0.02)	63.29	73%	Random
Asian	1.23 [1.07, 1.40]	2.94 (<0.01)	32.40	69%	Random
Caucasian	0.95 [0.84, 1.07]	0.85 (0.40)	7.46	33%	Random
AA vs AT + TT (dominant model)	1.17 [0.98, 1.39]	1.75 (0.08)	40.38	58%	Random
Asian	1.28 [1.01, 1.63]	2.02 (0.04)	25.37	61%	Random
Caucasian	0.92 [0.79, 1.09]	0.95 (0.34)	6.31	21%	Fixed
AA + AT vs TT (recessive model)	1.21 [1.03, 1.43]	2.36 (0.02)	58.32	71%	Random
Asian	1.32 [1.09, 1.59]	2.88 (<0.01)	29.51	66%	Random
Caucasian	0.90 [0.78, 1.03]	1.55 (0.12)	5.40	7%	Fixed
AA vs TT (homozygous comparison)	1.29 [1.02, 1.62]	2.17 (0.03)	56.20	70%	Random
Asian	1.46 [1.09, 1.95]	2.52 (0.01)	30.52	67	Random
Caucasian	0.87 [0.72, 1.05]	1.44 (0.15)	7.65	35 %	Fixed
AT vs TT (heterozygous comparison)	1.18 [1.01, 1.38]	2.14 (0.03)	47.23	64%	Random
Asian	1.27 [1.06, 1.52]	2.57 (0.01)	25.47	61%	Random
Caucasian	0.90 [0.78, 1.05]	1.33 (0.18)	3.68	0%	Fixed
**Precancerous lesions analysis (808 cases, 1,288 controls)**					
A allele vs T allele	1.09 [0.99, 1.20]	1.66 (0.08)	2.98	0%	Fixed
AA vs AT + TT (dominant model)	1.15 [0.85, 1.56]	0.90 (0.37)	1.92	0%	Fixed
AA + AT vs TT (recessive model)	1.21 [0.99, 1.49]	1.84 (0.07)	1.42	0%	Fixed
AA vs TT (homozygous comparison)	1.27 [0.91, 1.76]	1.42 (0.16)	2.37	0%	Fixed
AT vs TT (heterozygous comparison)	1.19 [0.96, 1.48]	1.59 (0.11)	0.69	0%	Fixed

### Overall analysis

**Figure 2:**
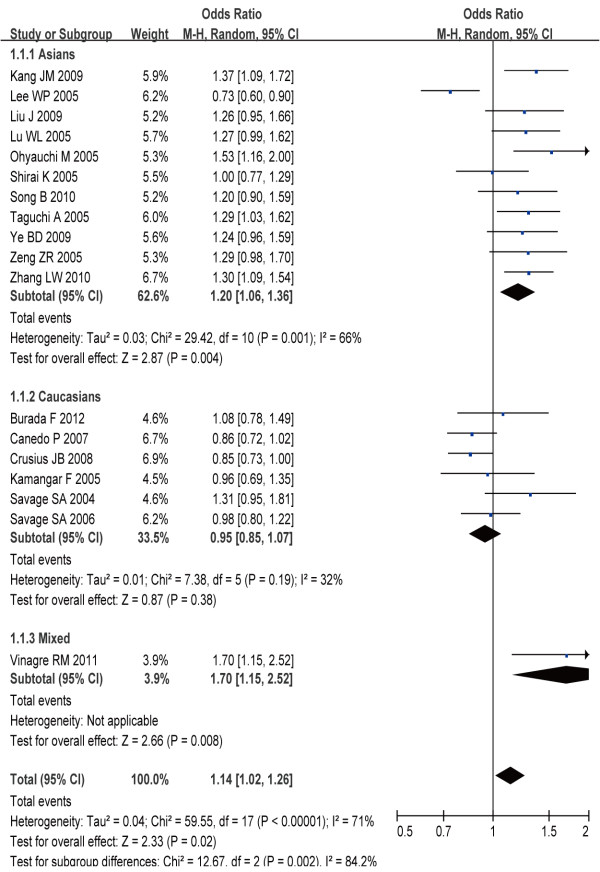
Overall OR for the association between IL-8-251A > T polymorphism and the risk of gastric carcinogenesis (allelic model).

Eighteen studies involved the correlations between IL-8-251 A > T polymorphism and gastric carcinogenesis. The heterogeneity obviously existed under most genetic models, which might be a result of the difference in ethnicity, country, source of controls and genotype methods, so random effects model was conducted to pool the results. By allelic comparison, A-allele genotypes were associated with gastric carcinogenesis, with a pooled OR of 1.14 (95% CI: 1.02–1.26, *P* = 0.02) (Figure 2). There were also significant associations in the recessive model (AA + AT versus TT) (OR = 1.18, 95% CI: 1.02–1.36, *P* = 0.03), and homozygous comparison (AA versus TT) (OR = 1.26, 95% CI: 1.02–1.57, *P* = 0.04), respectively, but not in the dominant model (AA vs AT + TT) (OR = 1.17, 95% CI = 0.98–1.38, *P* = 0.07) and the heterozygous comparison (AT versus TT) (OR = 1.14, 95% CI: 1.00–1.31, *P* = 0.05) (Table 2). In the stratified analysis by ethnicity, we found that gastric carcinogenesis risk was significant increased in Asian population under allele comparison (OR = 1.20, 95% CI: 1.06–1.36, *P* < 0.05), dominant model (OR = 1.28, 95% CI: 1.02–1.61, *P* = 0.04), recessive model (OR = 1.26, 95% CI: 1.08–1.47, *P* < 0.01), homozygous comparison (OR = 1.40, 95% CI: 1.08–1.83, *P* = 0.01), and heterozygous comparison (OR = 1.21, 95% CI: 1.09–1.35, *P* < 0.01). However, no significant association between this polymorphisms and gastric carcinogenesis risk was observed in all comparison models in Caucasians population (Table 2).

### Subgroup analysis

**Figure 3:**
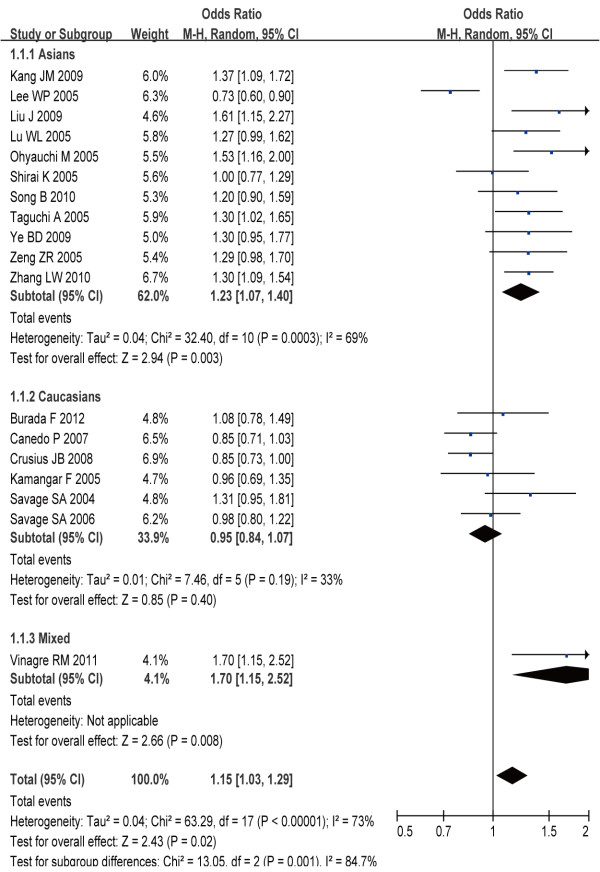
OR for the association between IL-8-251A > T polymorphism and the risk of gastric cancer (allelic model).

In the subgroup analysis, we evaluated the significance in patients with gastric cancer or precancerous lesion, respectively. In the gastric cancer group, we found that individuals with A-allele had significantly higher gastric cancer risks (OR = 1.15, 95% CI: 1.03–1.29, *P* = 0.02) (Figure 3). The results also indicated the significant risk under three models (recessive model: OR = 1.21, 95% CI: 1.03–1.43, *P* = 0.02; homozygous comparison: OR = 1.29, 95% CI: 1.02–1.62, *P* = 0.03; heterozygous comparison: OR = 1.18, 95% CI: 1.01–1.38, *P* = 0.03), whereas no significant risk was observed under dominant model (OR = 1.18, 95% CI: 1.01–1.38, *P* = 0.03). Moreover, we found that gastric cancer risk was significant increased in Asian population under allele comparison (OR = 1.23, 95% CI: 1.07–1.40, *P* < 0.01), dominant model (OR = 1.28, 95% CI: 1.01–1.63, *P* = 0.04), recessive model (OR = 1.32, 95% CI: 1.09–1.59, *P* < 0.01), homozygous comparison (OR = 1.46, 95% CI: 1.09–1.95, *P* = 0.01), and heterozygous comparison (OR = 1.27, 95% CI: 1.06–1.52, *P* = 0.01). However, no significant association between this polymorphisms and gastric cancer risk was observed in all comparison models in Caucasians population (Table 2).

**Figure 4:**
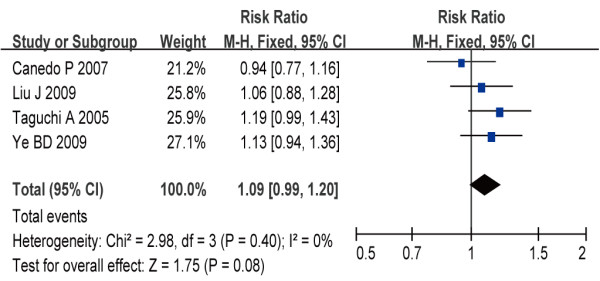
OR for the association between IL-8-251A > T polymorphism and the risk of gastric precancerous lesions (allelic model).

The information on the association between IL-8-251 A > T polymorphism and gastric precancerous lesions was available from four articles evaluated in this study (Figure 4). No remarkable association was presented between IL-8-251 A > T polymorphism and the presence of gastric precancerous lesions under the allele comparison (OR = 1.09, 95% CI: 0.99–1.20, *P* = 0.08), dominant model (OR = 1.15, 95% CI: 0.85–1.56, *P* = 0.37), recessive model (OR = 1.21, 95% CI: 0.99–1.49, *P* = 0.07), homozygous comparison (OR = 1.27, 95% CI: 0.91–1.76, *P* = 0.16), and heterozygous comparison(OR = 1.19, 95% CI: 0.96–1.48, *P* = 0.11).

### Publication bias

**Figure 5:**
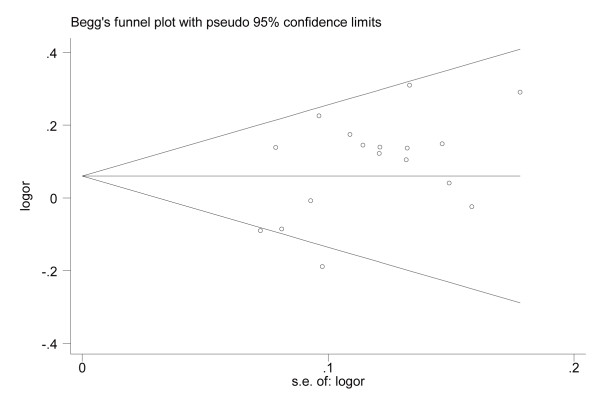
**Begger’s funnel plot of the meta-analysis of IL-8-251A > T polymorphism with gastric carcinogenesis under allele model.** Each point represents a separate study for the indicated association. Log[OR], natural logarithm of OR. Horizontal line, mean magnitude of the effect. Note: Funnel plot with pseudo 95% confidence limits was used.

Begger’s funnel plot and Egger’s linear regression test were performed to assess the publication bias of included studies. The shapes of the funnel plots seemed symmetrical in the allele comparison model (*P* = 0.484) (Figure 5). Egger’s test also did not show any significantly statistical evidence of publication bias under the allele comparison model (*P* =0.05), which indicated low risk of publication bias in this meta-analysis.

## Discussion

The pathogenesis of gastric carcinogenesis involves environmental factors, molecular signaling pathways, and host genetic factors [40]. The role of cytokine gene polymorphisms is currently a hot topic in gastric cancer research. Genome-wide association studies have identified several genetic loci associated with susceptibility to gastric carcinogenesis. Recently, a growing number of studies have suggested that SNPs of IL-8 251 allele, which is located at the promoter sequence of the IL-8 gene, might be associated with gastric tumorigenesis [41]. However, the results are contradictory. Hence, it is worth performing a meta-analysis of all eligible studies to investigate more precise estimation of this specific association.

The present meta-analysis summarized the correlation between the IL-8-251 A > T polymorphism and susceptibility to gastric carcinogenesis in 18 studies. The results suggested that the AA and AT genotypes of IL-8 -251A > T polymorphism appears to be associated with an overall increased risk of gastric carcinogenesis and is discovered as a risk factor of gastric cancer. Subgroup analysis by ethnicity allowed us to look for potential ethnic differences in the association. In the Asian population, the A-allele was associated with increased risk of gastric carcinogenesis and gastric cancer based on allele comparison, dominant model, recessive model, homozygote comparison, and heterozygous comparison. However, for the Caucasian population, IL-8 -251A > T polymorphism was not associated with increased risk of gastric carcinogenesis and gastric cancer. The A allele seemed to be dominant, but in the precancerous lesion subgroup, no significance was noted for all models. The possible reason could be that genetic risk factors differ between gastric cancer and precancerous lesions.The IL-8 promoter is estimated to be 1,500 bp. Several reports have shown relationship between IL-8 gene polymorphisms and human diseases, and all of them have focused on the A/T polymorphism at -251 upstream from the transcriptional start site. IL-8 -251A > T polymorphism has been associated with altered transcription levels of IL-8 by regulating the transcriptional activity of the gene and then proved to affect susceptibility to a large number of diseases. In the present analysis, we found an overall increase in gastric carcinogenesis of one or two allele variants as compared to wild allele T and homozygous TT genotype. After stratification into dominant and recessive genetic models, the dominant model (AA vs. AT + TT) (*P* = 0.07) and the recessive model (AA + AT vs. TT) (*P* = 0.02) both showed increased (1.18 fold) risk of gastric carcinogenesis. Even though the precise role of IL-8 -251A > T polymorphism in the development of gastric carcinogenesis is unknown, a plausible mechanism is that the mutations of IL-8 gene might increase gene transcription after binding to its high affinity cell surface receptor, which eventually attribute to the correlation between IL-8 and gastric carcinogenesis risk.

In fact, 18 studies were conducted as gastric cancer subgroup, whereas only 4 studies were conducted as precancerous lesions subgroup. We found that the pooled effect of studies of gastric cancer reported significant association between IL-8 -251A > T polymorphism and risk of gastric cancer. Moreover, the pooled effect of studies on patients with precancerous lesions did not show significant difference, the reasons for which might be deriving many elements and multiple mechanisms of gastric cancer and precancerous lesion disease. As is known, there are two mechanisms by which the gastric mucosa progresses to carcinoma, both starting from chronic gastritis. One mechanism is via precancerous lesions, such as gastric atrophy, intestinal metaplasia and adenomatous dysplasia leading to intestinal-type carcinomas characterized by glandular formation; the other is via hyperplastic or de novo changes leading to diffuse-type carcinomas characterized by isolated cancer cells with an infiltrative growth. However, our results showed that IL-8 -251A > T polymorphism was only associated with gastric cancer risk, but not with precancerous lesion. Thus, our meta-analysis suggested that genetic risk factors differ between gastric cancer and precancerous lesions.

In addition, results differed when stratifying the data by ethnicity. Associations between IL-8 -251A > T polymorphism and gastric carcinogenesis and gastric cancer were generally stronger in Asian than Caucasian population. This discrepancy from our meta-analysis may reflect the complex multifactorial etiology of gastric carcinogenesis.

Our results should be interpreted cautiously since some limitations exist in this present meta-analysis. First, only published studies were included in the meta-analysis. Therefore, the publication bias may have occurred, even though the use of a statistical test did not show it. Second, the number of included studies was relatively small with only about 5,321 cases. Moreover, other clinical factors such as age, ethics, and different chemotherapies in each study might lead to bias. Determining whether or not these factors influence the results of this meta-analysis would need further investigation. Third, the effect from our meta-analysis could be overestimated because many studies were retrospective cohort studies which had high risk of reporting biases. Therefore, more well-designed studies with large sample sizes are needed to further assess the precise effect of IL-8 -251A > T polymorphism in gastric carcinogenesis. Finally, the studies included in this meta-analysis were from different populations, it is possible that demographic factors can confound our results.

## Conclusions

Despite the limitations listed above, our meta-analysis results provide evidence that IL-8 -251A > T polymorphism is significantly associated with increased risk of gastric carcinogenesis, particularly in gastric cancer. Nevertheless, gastric carcinogenesis is a multifactorial and multistep process, so our results should be examined cautiously by an adequately designed prospective studies, and larger clinical trials with widely accepted assessment methods.

## Materials and methods

### Search strategy

A systematic literature search of MEDLINE (updated to June, 2013), Embase (updated to June, 2013), and web of Science (updated to June, 2013), and CNKI (Chinese National Knowledge Infrastructure) databases was conducted by two study investigators (D.C. and Y. H.) independently for all relevant articles. Key words used in the research included “Interleukin-8”, “IL-8”, “CXCL8”, “gastric cancer”, “stomach cancer”, “precancerous lesion”, “polymorphism”, “SNP”, “gene variant”, “gene mutation”, and “gastric tumor”.

### Inclusion and exclusion criteria

Studies eligible for inclusion in this meta-analysis should meet the following criteria: (a) case–control studies or cohort studies focused on association between IL-8-251 A > T polymorphism and risk of gastric carcinogenesis; (b) patients have pathologically or histological confirmed gastric cancer and/or precancerous lesions; (c) The studies provided the number of cases and controls for various genotypes. The exclusion criteria of the meta-analysis were: (a) animal studies; (b) meta-analyses, letters, reviews or editorial comments; (c) studies with duplicate data or incomplete date. When an individual author published several articles obtained from the same patient population, only the newest or most complete article was included in the analysis.

### Data extraction

Information was carefully extracted from all the eligible publications. The following data were collected from each study: first author’s name, publication date, country, ethnicity, source of controls, genotyping method, total numbers of cases and controls, number of cases and controls for each IL-8-251 A > T polymorphism, and *P* value for HWE. An attempt was made to contact authors if data presentation was incomplete or if it was necessary to resolve an apparent conflict or inconsistency in the article. Any disagreements were resolved by consensus.

### Statistical analysis

Review manager 5.1 program provided by the Cochrane Library and Stata (Version12.0, Stata Corporation) were used to perform all the statistical analysis. The association was evaluated with the use of the allelic comparison (A versus T), as well as the dominant model (AA versus AT + TT), the recessive model (AA + AT versus TT), the homozygous comparison (AA versus TT), and heterozygous comparison (AT versus TT), respectively. Two models of pooling data for dichotomous outcomes were conducted: the random-effects model and the fixed-effects model. The pooled statistical analysis was calculated using the fixed effects model, but a random-effect model was performed when the *P* value of heterogeneity test was <0.1 (or I^2^ > 50%). The odds ratio (OR) and 95% confidence interval (CI) were calculated for each study, and the combined OR and 95% CI were calculated for all eligible studies. OR was the proportion of the exposed population in whom disease has developed over the proportion of the unexposed population in whom disease has developed in a case–control study. The significance of the combined OR was determined by the Z-test, in which *P* < 0.05 was considered significant. Heterogeneity assumption was assessed by the chisquare based Q test and was regarded to be statistically significant if *P* < 0.10. The potential publication bias was assessed by Begg’s funnel plot and Egger’s test [42,43].

## Abbreviations

IL-8: Interleukin-8; OR: Odds ratio.

## Competing interests

The authors declare that they have no competing interests.

## Authors’ contributions

DC conceived and designed the study. DC and YH drafted the manuscript. WZ and YM collected the data. DC performed the data analysis. All authors have read and approved the final manuscript.

## References

[B1] Kamangar F Dores GM Anderson WF Patterns of cancer incidence, mortality, and prevalence across five continents: defining priorities to reduce cancer disparities in different geographic regions of the world J clin oncol : offic j American Soc Clin Oncol 2006 24 14 2137 2150 10.1200/JCO.2005.05.2308 16682732

[B2] Parkin DM Bray F Ferlay J Pisani P Global cancer statistics, 2002 CA Cancer J Clin 2005 55 2 74 108 15761078 10.3322/canjclin.55.2.74

[B3] Krejs GJ Gastric cancer: epidemiology and risk factors Dig Dis 2010 28 4–5 600 603 21088409 10.1159/000320277

[B4] Stoicov C Saffari R Cai X Hasyagar C Houghton J Molecular biology of gastric cancer: Helicobacter infection and gastric adenocarcinoma: bacterial and host factors responsible for altered growth signaling Gene 2004 341 1 17 15474284 10.1016/j.gene.2004.07.023

[B5] Caldeira J Simoes-Correia J Paredes J Pinto MT Sousa S Corso G Marrelli D Roviello F Pereira PS Weil D CPEB1, a novel gene silenced in gastric cancer: a drosophila approach Gut 2012 61 8 1115 1123 22052064 10.1136/gutjnl-2011-300427

[B6] Todorovic-Rakovic N Milovanovic J Interleukin-8 in Breast Cancer Progression J Interferon Cytokine Res: offic j Intl Soc Interferon Cytokine Res 2013 30 10 563 570 10.1089/jir.2013.0023 PMC3793647 23697558

[B7] Keeley EC Mehrad B Strieter RM CXC chemokines in cancer angiogenesis and metastases Adv Cancer Res 2010 106 91 111 20399957 10.1016/S0065-230X(10)06003-3 PMC3069502

[B8] Xie K Interleukin-8 and human cancer biology Cytokine and Growth Factor Research 2001 12 4 375 391 10.1016/s1359-6101(01)00016-8 11544106

[B9] Singh RK Varney ML IL-8 expression in malignant melanoma: implications in growth and metastasis Histol Histopathol 2000 15 3 843 849 10963128 10.14670/HH-15.843

[B10] Zuccari DA Leonel C Castro R Gelaleti GB Jardim BV Moscheta MG Regiani VR Ferreira LC Lopes JR Neto Dde S An immunohistochemical study of interleukin-8 (IL-8) in breast cancer Acta histochemica 2012 114 6 571 576 22244449 10.1016/j.acthis.2011.10.007

[B11] Browne A Sriraksa R Guney T Rama N Van Noorden S Curry E Gabra H Stronach E El-Bahrawy M Differential expression of IL-8 and IL-8 receptors in benign, borderline and malignant ovarian epithelial tumours Cytokine 2013 64 1 413 421 23727325 10.1016/j.cyto.2013.05.006

[B12] Michaud DS Daugherty SE Berndt SI Platz EA Yeager M Crawford ED Hsing A Huang WY Hayes RB Genetic polymorphisms of interleukin-1B (IL-1B), IL-6, IL-8, and IL-10 and risk of prostate cancer Cancer res 2006 66 8 4525 4530 16618781 10.1158/0008-5472.CAN-05-3987

[B13] Ewington L Taylor A Sriraksa R Horimoto Y Lam EW El-Bahrawy MA The expression of interleukin-8 and interleukin-8 receptors in endometrial carcinoma Cytokine 2012 59 2 417 422 22626766 10.1016/j.cyto.2012.04.036

[B14] Lee YS Choi I Ning Y Kim NY Khatchadourian V Yang D Chung HK Choi D LaBonte MJ Ladner RD Interleukin-8 and its receptor CXCR2 in the tumour microenvironment promote colon cancer growth, progression and metastasis BJC 2012 106 11 1833 1841 22617157 10.1038/bjc.2012.177 PMC3364111

[B15] Ju D Sun D Xiu L Meng X Zhang C Wei P Interleukin-8 is associated with adhesion, migration and invasion in human gastric cancer SCG-7901 cells Med Oncol 2012 29 1 91 99 21191670 10.1007/s12032-010-9780-0

[B16] Hull J Rowlands K Lockhart E Sharland M Moore C Hanchard N Kwiatkowski DP Haplotype mapping of the bronchiolitis susceptibility locus near IL8 Hum Genet 2004 114 3 272 279 14605870 10.1007/s00439-003-1038-x

[B17] McCarron SL Edwards S Evans PR Gibbs R Dearnaley DP Dowe A Southgate C Easton DF Eeles RA Howell WM Influence of cytokine gene polymorphisms on the development of prostate cancer Cancer res 2002 62 12 3369 3372 12067976

[B18] Huang Q Wang C Qiu LJ Shao F Yu JH IL-8-251A > T polymorphism is associated with breast cancer risk: a meta-analysis J Cancer Res Clin Oncol 2011 137 7 1147 1150 21468699 10.1007/s00432-011-0981-5 PMC11828166

[B19] Wang Z Wang C Zhao Z Liu F Guan X Lin X Zhang L Association between -251A > T polymorphism in the interleukin-8 gene and oral cancer risk: a meta-analysis Gene 2013 522 2 168 176 23545310 10.1016/j.gene.2013.03.066

[B20] Landi S Moreno V Gioia-Patricola L Guino E Navarro M De Oca J Capella G Canzian F Association of common polymorphisms in inflammatory genes interleukin (IL)6, IL8, tumor necrosis factor alpha, NFKB1, and peroxisome proliferator-activated receptor gamma with colorectal cancer Cancer res 2003 63 13 3560 3566 12839942

[B21] van der Kuyl AC Polstra AM Weverling GJ Zorgdrager F van den Burg R Cornelissen M An IL-8 gene promoter polymorphism is associated with the risk of the development of AIDS-related Kaposi’s sarcoma: a case–control study AIDS 2004 18 8 1206 1208 15166538 10.1097/00002030-200405210-00016

[B22] Burada F Angelescu C Mitrut P Ciurea T Cruce M Saftoiu A Ioana M Interleukin-4 receptor -3223T → C polymorphism is associated with increased gastric adenocarcinoma risk Can J Gastroenterol = Journal canadien de gastroenterologie 2012 26 8 532 536 10.1155/2012/804173 PMC3414475 22891178

[B23] Canedo P Castanheira-Vale AJ Lunet N Pereira F Figueiredo C Gioia-Patricola L Canzian F Moreira H Suriano G Barros H The interleukin-8-251*T/*A polymorphism is not associated with risk for gastric carcinoma development in a Portuguese population Eur J Cancer Prev 2008 17 1 28 32 18090907 10.1097/CEJ.0b013e32809b4d0f

[B24] Crusius JB Canzian F Capella G Pena AS Pera G Sala N Agudo A Rico F Del Giudice G Palli D Cytokine gene polymorphisms and the risk of adenocarcinoma of the stomach in the European prospective investigation into cancer and nutrition (EPIC-EURGAST) Ann Oncol: offic J Eur Soc Med Oncol / ESMO 2008 19 11 1894 1902 10.1093/annonc/mdn400 18628242

[B25] Kamangar F Abnet CC Hutchinson AA Newschaffer CJ Helzlsouer K Shugart YY Pietinen P Dawsey SM Albanes D Virtamo J Polymorphisms in inflammation-related genes and risk of gastric cancer (Finland) Cancer causes & control: CCC 2006 17 1 117 125 16411061 10.1007/s10552-005-0439-7

[B26] Kang JM Kim N Lee DH Park JH Lee MK Kim JS Jung HC Song IS The effects of genetic polymorphisms of IL-6, IL-8, and IL-10 on Helicobacter pylori-induced gastroduodenal diseases in Korea J Clin Gastroenterol 2009 43 5 420 428 19077731 10.1097/MCG.0b013e318178d1d3

[B27] Lee WP Tai DI Lan KH Li AF Hsu HC Lin EJ Lin YP Sheu ML Li CP Chang FY The -251T allele of the interleukin-8 promoter is associated with increased risk of gastric carcinoma featuring diffuse-type histopathology in Chinese population Clin cancer res: an offic Jo Am Assoc Cancer Res 2005 11 18 6431 6441 10.1158/1078-0432.CCR-05-0942 16166417

[B28] Liu J Xing CZ Sun LP Gong YH Bai XL Zhang Y Chen W Yuan Y The relationship of interleukin-8-251 with gastric cancer and precancerous lesion Chin J Dig endoscopy 2009 26 6 310 312

[B29] Lu W Pan K Zhang L Lin D Miao X You W Genetic polymorphisms of interleukin (IL)-1B, IL-1RN, IL-8, IL-10 and tumor necrosis factor alpha and risk of gastric cancer in a Chinese population Carcinogenesis 2005 26 3 631 636 15579481 10.1093/carcin/bgh349

[B30] Ohyauchi M Imatani A Yonechi M Asano N Miura A Iijima K Koike T Sekine H Ohara S Shimosegawa T The polymorphism interleukin 8–251 A/T influences the susceptibility of helicobacter pylori related gastric diseases in the Japanese population Gut 2005 54 3 330 335 15710978 10.1136/gut.2003.033050 PMC1774396

[B31] Savage SA Abnet CC Mark SD Qiao YL Dong ZW Dawsey SM Taylor PR Chanock SJ Variants of the IL8 and IL8RB genes and risk for gastric cardia adenocarcinoma and esophageal squamous cell carcinoma Cancer epid, biomarkers & prevention : Pub Am Assoc Cancer Res, cosponsored by the Am Soc Prev Oncol 2004 13 12 2251 2257 15598788

[B32] Savage SA Hou L Lissowska J Chow WH Zatonski W Chanock SJ Yeager M Interleukin-8 polymorphisms are not associated with gastric cancer risk in a polish population Cancer epid, biomarkers & prevention: Pub Am Assoc Cancer Res, cosponsored by the Am Soc Prev Oncol 2006 15 3 589 591 10.1158/1055-9965.EPI-05-0887 16537722

[B33] Shirai K Ohmiya N Taguchi A Mabuchi N Yatsuya H Itoh A Hirooka Y Niwa Y Mori N Goto H Interleukin-8 gene polymorphism associated with susceptibility to non-cardia gastric carcinoma with microsatellite instability J Gastroenterol Hepatol 2006 21 7 1129 1135 16824064 10.1111/j.1440-1746.2006.04443.x

[B34] Song B Zhang D Wang S Zheng H Wang X Association of interleukin-8 with cachexia from patients with low-third gastric cancer Comparative and functional genomics 2009 212345 10.1155/2009/212345 PMC2796459 20037740

[B35] Taguchi A Ohmiya N Shirai K Mabuchi N Itoh A Hirooka Y Niwa Y Goto H Interleukin-8 promoter polymorphism increases the risk of atrophic gastritis and gastric cancer in Japan Cancer epid, biomarkers & prevention: Pub Am Assoc Cancer Res, cosponsored by the Am Soc Prev Oncol 2005 14 11 Pt 1 2487 2493 10.1158/1055-9965.EPI-05-0326 16284368

[B36] Vinagre RM Corvelo TC Arnaud VC Leite AC Barile KA Martins LC Determination of strains of Helicobacter pylori and of polymorphism in the interleukin-8 gene in patients with stomach cancer Arquivos de gastroenterologia 2011 48 1 46 51 21537542 10.1590/s0004-28032011000100010

[B37] Ye BD Kim SG Park JH Kim JS Jung HC Song IS The interleukin-8-251 A allele is associated with increased risk of noncardia gastric adenocarcinoma in Helicobacter pylori-infected Koreans J Clin Gastroenterol 2009 43 3 233 239 18542040 10.1097/MCG.0b013e3181646701

[B38] Zeng ZR Zhou SZ Liao SY Chen B Li CJ Chen HH Hu PJ Correlation of polymorphism of interleukin 8 gene-251 locus and gastric cancer in high and low prevalence regions in China J Sun Yat-Sen Uni (Med Sci) 2005 26 5 537 540

[B39] Zhang L Du C Guo X Yuan L Niu W Yu W Er L Wang S Interleukin-8-251A/T polymorphism and Helicobacter pylori infection influence risk for the development of gastric cardiac adenocarcinoma in a high-incidence area of China Mol Biol Rep 2010 37 8 3983 3989 20300863 10.1007/s11033-010-0057-7

[B40] Bornschein J Malfertheiner P Gastric carcinogenesis Langenbeck’s archives of surgery/Deutsche Gesellschaft fur Chirurgie 2011 396 6 729 742 10.1007/s00423-011-0810-y 21611816

[B41] Wang J Pan HF Hu YT Zhu Y He Q Polymorphism of IL-8 in 251 allele and gastric cancer susceptibility: a meta-analysis Dig Dis Sci 2010 55 7 1818 1823 19777350 10.1007/s10620-009-0978-y

[B42] Begg CB Mazumdar M Operating characteristics of a rank correlation test for publication bias Biometrics 1994 50 4 1088 1101 7786990

[B43] Egger M Davey Smith G Schneider M Minder C Bias in meta-analysis detected by a simple, graphical test BMJ 1997 315 7109 629 634 9310563 10.1136/bmj.315.7109.629 PMC2127453

